# A Repeatable Motion Scheme for Kinematic Control of Redundant Manipulators

**DOI:** 10.1155/2019/5426986

**Published:** 2019-09-18

**Authors:** Kong Ying, Tang Qingqing, Zhang Ruiyang, Ye Lv

**Affiliations:** Department of Information and Electronic Engineering, Zhejiang University of Science and Technology, Hangzhou, China

## Abstract

To achieve closed trajectory motion planning of redundant manipulators, each joint angle has to be returned to its initial position. Most of the repeatable motion schemes have been proposed to solve kinematic problems considering only the initial desired position of each joint at first. Actually, it is very difficult for various joint angles of the robot arms to be positioned in the expected trajectory before moving. To construct an effective kinematic model, a novel optimal programming index based on a recurrent neural network is designed and analyzed in this paper. The repetitiveness and timeliness are presented and analyzed. Combining the kinematic equation constraint of manipulators, a repeatable motion scheme is formulated. In addition, the Lagrange multiplier theorem is introduced to prove that such a repeatable motion scheme can be converted into a time-varying linear equation. A finite-time neural network solver is constructed for the solution of the motion scheme. Simulation results for two different trajectories illustrate the accuracy and timeliness of the proposed motion scheme. Finally, two different repetitive schemes are compared and verified the optimal time for the novelty of the proposed kinematic scheme.

## 1. Introduction

Robot manipulators have been playing an important role in various kinds of engineering fields. They have been widely used to perform effective and high-intensive repetitive work, such as car assembling, logistics handling, and sculpturing [1–3]. Robot arms usually have two types in needs of special operators. One is the redundant manipulators which have more degrees of freedom (DOF) than the given tasks required while the other one is the ordinary nonredundant manipulators which can complete the objective directly. Correspondingly, nonredundant manipulators refer to the one that has no additional DOF when performing a given task. Redundant manipulators are more flexible and advantageous for its redundant DOF. That is the reason why it is increasingly important in practical engineering applications [4, 5].

One of the fundamental problems in redundant manipulators motion controlling is redundancy solutions, known as inverse kinematics, which attracts many researchers' interests [6, 7]. Given the position and pose of the end-effector, calculating the homologous trajectories of joint angles with the redundant manipulators is named inverse kinematics. Liegeios-Chauvel et al. put forward a gradient projection method based on inverse kinematics solution to divide the particular motion controlling into zero space by solving the optimization goal to regulate the solutions for redundancy [8]. From then on, many researchers study pseudo inverse approaches for kinematic equation of redundant manipulators [9, 10]. However, these approaches do not only take a heavy burden of calculations, but also require the Jacobian of manipulators to be full rank, which is hard to realize.

With the development of neural networks, recurrent neural networks based on negative gradient directions are emerged. Due to the high efficient ability for computing, gradient neural networks (GNNs) are widely used in identifications and matrix equations [10]. GNN is set up by establishing non-negative direction function and to obtain a scheme for kinematic controlling. When applied to motion planning of redundant manipulators, the convergent errors generated by GNN are always lagged behind the ideal one. That is, every joint angle of manipulators cannot return to their initial position in the end, which may cause nonrepetition phenomenon in trajectory planning of the robot manipulators. With the deepening of research studies in repeatable controlling of redundant manipulators, various velocity schemes based on online quadratic optimization have been developed. Such optimal schemes incorporate the equality and inequality constraints and avoid the limitation of joint angles and joint velocities. For efficient repetitive tasks, Zhang et al. firstly introduced a repetitive motion index as the optimization criterion, using Zhang neural network (ZNN) to solve the redundancy problems [11–13]. Then, various motion schemes combining the physical joint constraints are formulated as an optimal programming index subjected to the kinematic equation of manipulators. In addition, theoretical analyses prove that the motion schemes can be converted into time-varying equations by using the Lagrange multiplier. When considering necessary conditions of Karush-Kuhn-Tucker (KKT) for nonlinear optimization problems, such an optimal programming index also can be converted into linear variational inequality (LVI) and a piecewise linear projection equation (PLPE) [14, 15]. Various neural networks have spung up to solve the LVI and PLPE. Simulations on different types of redundant manipulators are studied and different shapes of the trajectory tasks are given, which verified the effectiveness and superiority of the proposed optimal programming index for repeatable motion planning as well as the corresponding neural solvers [16, 17].

Although, most of the aforementioned approaches for repeatable motion planning of redundant manipulators are effective, the convergent time of the dynamical equations has not been ensured. That is the optimal programming index for motion controlling using neural solvers can make the joint angles of the manipulators back to initial desired position as long as time goes infinity. For the perspective of finite-time convergence, Li et al. first proposed a finite-time neural network (FTNN) model to solve the repetitive motion scheme based on ZNN in order to ensure that the convergent time is finite [18]. Then, around this FTNN, various revised FTNN models are constructed to accelerate the convergent rate of ZNN [19–21]. In the literature [22], a motion scheme of mobile robot arms based on finite-time convergence property has been set up to apply in the grasping work of the manipulators.

A redundant manipulator is a part of a robot. It is hard to locate every joint angle in the desired state at first. It is not efficient for adjusting the positions of joint angles through self-movement. However, most of the above repetitive motion plans and different neural solvers, which are mainly for ideal initial state, do not consider the deviations of manipulators. These models of repetitive motion planning based on pseudo inverse and asymptotic convergent dynamic recurrent neural networks have been studied by many researchers. Few studies are reported for finite-time repeatable motion controlling when the joint angles are deviated from the initial desired position at first [23]. In the literature [24], only a new type of terminal neural network is researched for solving motion scheme of ZNN, which has been designed from the perspective of controlling. In [25], initial deviations of joint angles are considered. The optimization performance indices is still based on infinite-time convergence, and only the neural solver for motion scheme is designed for finite-time convergence.

The remainder of this paper is organized as follows. The kinematic equations of redundant manipulators are established in Section 2.  Section 3 gives out the optimal programming index, and a repeatable motion scheme of manipulators is formulated and analyzed. In Section 4, a terminal recurrent neural network algorithm is proposed to solve the mentioned motion scheme. In Section 5, simulation results on two different path trajectories with Katana6M180 manipulators verified the effectiveness and superiority of the repeatable motion scheme and the terminal neural solver. Finally, remarks and conclusions are presented in Section 6. The main contributions of this paper are summarized in the following aspects:

1. A new optimal programming index for repeatable motion planning of redundant manipulators is exploited. It is the first time to use this performance index, which can ensure the joint angles back to their initial desired positions in finite time no matter considering the initial state of each joint of the manipulators.
2. A special kind of neural model based on recurrent neural networks is presented to solve the repeatable motion scheme. The activation of the neural solvers has adopted limited-value function, which is applicable in practical application problems.
3. Two different tracking tasks with redundant manipulator Katana6M180 are introduced to illustrate the superiority and effectiveness of the proposed repeatable motion scheme. Comparison results of various repetitive motion schemes are visualized in the end.

## 2. Kinematic Structure of Katana6M180 Robot Arm

**Figure 1:**
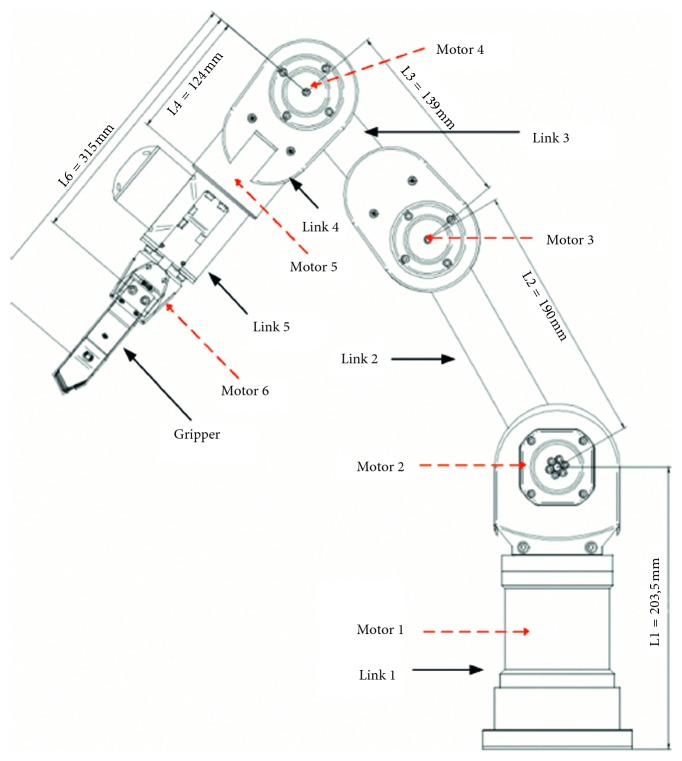
Design structure of redundant manipulator Katana6M180.

In this section, a redundant manipulator Katana6M180 model has been set up for illustrating the effectiveness of the proposed repeatable motion scheme. The mechanical structure of the Katana6M180 robot arm is shown in Figure 1. The Katana6M180 robot arm is composed of five degrees of freedom (DOF) with three-DOF elbow for revolute joints, two-DOF wrist for revolute joints, and a gripper connected to the end-effector. The range of angular motion of Katana6M180 is displayed in Table 1. From the Table 1, joint *θ*_1_ is the angle between horizontal line and link 1 (*l*_1_), joint *θ*_2_ is the angle between link 2 and link 3, joint *θ*_3_ is the angle between link 3 and link 4, and joint *θ*_4_ is the angle between link 4 and link 5. Joint *θ*_5_ is between motor 5 and motor 6. In Table 1, it is shown that the rotation and extension of the joint angles are limited by the mechanical arm itself.

### 2.1. Kinematic Foundation of Katana6M180 Robot Arm

**Figure 2:**
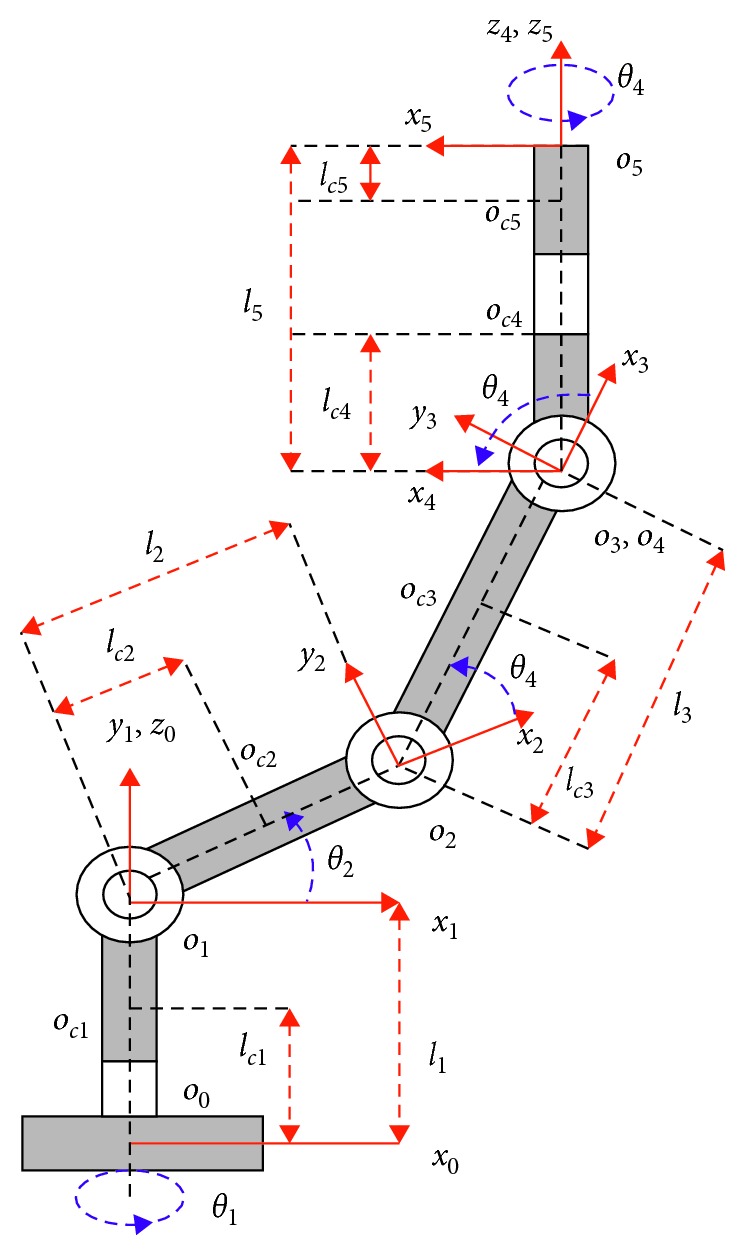
Mechanical assignment for Katana6M180 manipulator.

In this section, a kinematic frame for Katana6M180 is formulated with the DH parameters, homogeneous matrix, transformation matrix, and Jacobian matrix. The kinematic redundant arm of Katana6M180 is shown in Figure 2. From Figure 2, *z*_4_ and *z*_5_ are parallel to each other and are vertical to *z*_3_. *z*_4_ is orthogonal to *x*_4_ and *x*_5_. The motion point *o*_4_ in the frame of *o*_4_ *x*_4_ *y*_4_ *z*_4_ is chosen to work in coordination with *o*_3_, which is the original point of *o*_3_ *x*_3_ *y*_3_ *z*_3_. The length of *a*_4_ is zero. From the end of the end gripper, the turning point *o*_5_ of the frame *o*_5_ *x*_5_ *y*_5_ *z*_5_ is fixed at the end of link 5. *y*_4_ is supposed to be the center of the right-handed frames. The D-H parameters are shown in Table 2.

The homogeneous transformation matrix for K6M180 is

(1) $$\begin{array}{c} T_{5}^{0}=\left(\begin{array}{cccc} T_{11} & T_{12} & T_{13} & T_{14}\\ T_{21} & T_{22} & T_{23} & T_{24}\\ T_{31} & T_{32} & T_{33} & T_{34}\\ T_{41} & T_{42} & T_{43} & T_{44} \end{array}\right), \end{array}$$

where *T*_11_=*C*_1_ *C*_23_ *C*_5_+*S*_1_ *S*_5_, *T*_21_=*C*_234_ *C*_5_ *S*_1_ − *C*_1_ *S*_5_, *T*_12_=*C*_5_ *S*_1_ − *C*_1_ *C*_234_ *S*_5_, *T*_22_=−*C*_1_ *C*_5_ − *C*_234_ *S*_1_ *S*_5_, *T*_13_=*C*_1_ *S*_234_, *T*_23_=*S*_1_ *S*_234_, *T*_14_=*C*_1_(*l*_2_ *C*_2_+*l*_3_ *C*_23_+(*l*_4_+*l*_5_)*S*_234_), *T*_24_=*S*_1_(*l*_2_ *C*_2_+*l*_3_ *C*_23_+(*l*_4_+*l*_5_)*S*_234_), *T*_31_=*C*_5_ *S*_234_, *T*_41_=0, *T*_32_=−*S*_234_ *S*_5_, *T*_42_=0, *T*_33_=−*C*_234_,  and *T*_34_=*l*_1_ − (*l*_4_+*l*_5_)*C*_234_+*l*_2_ *S*_2_+*l*_3_ *S*_23_. Moreover, *C*_*ijk*_ is the simplified notation for cos(*θ*_*i*_+*θ*_*j*_+*θ*_*k*_). *S*_*ijk*_ is the simplified notation for sin(*θ*_*i*_+*θ*_*j*_+*θ*_*k*_). Considering the position of the end-effector, the *x*, *y*, *z* compose the three dimensions of the following position vector *r*_5_^0^ of the K6M180 frame:

(2) $$\begin{array}{c} r_{5}^{0}=\left[\begin{array}{c} C_{1}\left(l_{2}C_{2}+l_{3}C_{23}+\left(l_{4}+l_{5}\right)S_{234}\right)\\ S_{1}\left(l_{2}C_{2}+l_{3}C_{23}+\left(l_{4}+l_{5}\right)S_{234}\right)\\ l_{1}-\left(l_{4}+l_{5}\right)C_{234}+l_{2}S_{2}+l_{3}S_{23} \end{array}\right]. \end{array}$$

The solutions for inverse kinematic problems are not suitable for using forward kinematic formations. For the existence of singularity with the robotic arms, it is necessary to find out an appropriate kinematic solution to solve the inverse kinematics.

### 2.2. Traditional Solution for Inverse Kinematic of Katana6M180 Robot Arm

Given a special trajectory with the end-effector of the Katana6M180 manipulator, the inverse problem is to solve each joint angle corresponding to the moving trail of the end-effector in the dimensional space. For the solving method for redundant manipulators, algebraic algorithms, iterative algorithms, and geometric algorithms are the primary solutions for trajectory planning problems. Algebraic algorithms do not propose a systematic method for choosing a special solution of the possible ways for motion planning of redundant arms. Iterative algorithms take a burden of the computational time, and in the singularity situations, experimental error may not reach to zero in a long time.

## 3. Repeatable Motion Scheme for Redundant Manipulators

As mentioned above, given a desired trajectory for redundant manipulators, each joint angle of the robot arm has to be returned to its desired initial position in the end. Traditional algorithms for inverse motion designing are not applicable for repetitive control especially in the situation that the initial position of the manipulators may not be in their desired places at first. Furthermore, the joint constraints of redundant manipulators should be taken into account. Motivated by these practical ideas, a repeatable inverse motion scheme for redundant manipulators is formulated as follows:

(3) $$\begin{array}{c} \begin{array}{cc} \text{minimize} & \frac{1}{2}{\left(\dot{\theta }\left(t\right)+\beta_{\theta }c\right)}^{T}\left(\dot{\theta }\left(t\right)+\beta_{\theta }c\right)\\ \text{subject to} & J\left(\theta \right)\dot{\theta }={\dot{r}}^{*}+k_{p}\left(r^{*}-f\left(\theta \right)\right), \end{array} \end{array}$$

where *c*=|*θ*(*t*) − *θ* ^*∗*^(0)|^*α*^sgn(*θ*(*t*) − *θ* ^*∗*^(0)). 0 < *α* < 1, *θ* ^*∗*^(0) is the desired initial position of the joint variable vector. The design parameter *β*_*θ*_ > 0 is used to form the joint displacement of the manipulators. *k*_*p*_ > 0 ∈ *R* represents the magnitude parameter to control the convergent speed of the end-effector. *r* ^*∗*^ is the desired motion trajectory of the end-effector. ${\dot{r}}^{*}$ is the speed vector of the end-effector. Considering that the initial position of the end-effector may not be at the desired initial point, the position error between the actual trajectory *f*(*θ*) and the desired motion trajectory *r* ^*∗*^ are needed to be reduced for changing the motion direction.

### 3.1. Convergent Analysis

According to the ZNN theories, the following equation has been formulated:

(4) $$\begin{array}{c} \dot{E}\left(t\right)=-\mu E\left(t\right), \end{array}$$

where *E*(*t*)=*θ*_*t*_ − *θ*_0_, *μ* > 0. It follows the fact that the convergent error *E*(*t*) reduces to zero as time goes by. By applying the ZNN theory, we get the scheme $\dot{\theta }\left(t\right)=-\mu \left(\theta \left(t\right)-\theta \left(0\right)\right)$, then, $\dot{\theta }\left(t\right)+\mu \left(\theta \left(t\right)-\theta \left(0\right)\right)=0$. Therefore, we obtain the ZNN repetitive index:

(5) $$\begin{array}{c} \frac{1}{2}{\left(\dot{\theta }\left(t\right)+\mu \left(\theta \left(t\right)-\theta \left(0\right)\right)\right)}^{T}\left(\dot{\theta }\left(t\right)+\mu \left(\theta \left(t\right)-\theta \left(0\right)\right)\right). \end{array}$$

Motivated by the ZNN theory, a finite-time convergent neural network model has been proposed, which greatly reduced the convergent time. The error dynamics of the finite-time convergence neural model is described as follows:

(6) $$\begin{array}{c} \dot{E}\left(t\right)=-\beta_{\theta }{\left|E\left(t\right)\right|}^{\alpha }\mathrm{sgn}\left(E\left(t\right)\right). \end{array}$$

Setting the following Lyapunov function,

(7) $$\begin{array}{c} V\left(t\right)=\frac{1}{2}\;E^{2}\left(t\right). \end{array}$$

The derivation of equation (7) with respect to time is as follows:

(8) $$\begin{array}{c} \dot{V}\left(t\right)=-\beta_{\theta }{\left|E\left(t\right)\right|}^{\alpha }\left|E\left(t\right)\right|=-\beta_{\theta }\left({\sqrt{2V\left(t\right)}}^{1+\alpha }\right)<0. \end{array}$$

For $\dot{V}\left(t\right)<0$, the finite-time convergent model (6) is asymptotically stable in the end. In addition, the above equation can be rewritten as

(9) $$\begin{array}{c} \frac{1}{V^{1+\alpha /2}\left(t\right)}dV\left(t\right)=-\beta_{\theta }2^{\left(1+\alpha \right)/2}dt\;. \end{array}$$

Integrating both sides produces

(10) $$\begin{array}{c} \frac{2}{1-\alpha }\;V^{\left(1-\alpha \right)/2}\left(t\right)-\frac{2}{1-\alpha }V^{\left(1-\alpha \right)/2}\left(0\right)=-\beta_{\theta }2^{\left(1+\alpha \right)/2}t. \end{array}$$

When *E*(*t*) has been converged to zero, *V*(*t*)=0, *V* ^(1 − *α*)/2^(*t*)=0, equation (8) is rewritten as follows:

(11) $$\begin{array}{c} t_{s}=\frac{2^{\left(1-\alpha \right)/2}}{\beta_{\theta }\left(1-\alpha \right)}V^{\left(1-\alpha \right)/2}\left(0\right). \end{array}$$

For repetitive motion planning of redundant manipulators, we set *E*(*t*)=*θ*(*t*) − *θ* ^*∗*^(0), and the dynamical equation (6) can be changed into (11).

(12) $$\begin{array}{c} \dot{\theta }\left(t\right)+\beta_{\theta }{\left|\theta \left(t\right)-\theta^{*}\left(0\right)\right|}^{\alpha }\mathrm{sgn}\left(\theta \left(t\right)-\theta^{*}\left(0\right)\right)=0. \end{array}$$

That is,

(13) $$\begin{array}{c} {\left\|\dot{\theta }\left(t\right)+\beta_{\theta }{\left|\theta \left(t\right)-\theta^{*}\left(0\right)\right|}^{\alpha }\mathrm{sgn}\left(\theta \left(t\right)-\theta^{*}\left(0\right)\right)\right\|}_{2}^{2}=0. \end{array}$$

According to the solution of norm problem (11), we set the repeatable motion index in equation (3).

### 3.2. Scheme Formulation

For the repeatable motion scheme (3), the performance kinematic index is reformulated as $\left(\dot{\theta }\left(t\right)+\beta_{\theta }c\right)\left(\dot{\theta }\left(t\right)+\beta_{\theta }c\right)=\dot{\theta }{\left(t\right)}^{T}\dot{\theta }\left(t\right)+2\beta_{\theta }c^{T}\dot{\theta }\left(t\right)+\beta_{\theta }c^{T}\beta_{\theta }c$. Since the motion scheme is only considered the velocity level and $\dot{\theta }\left(t\right)$ is the variable vector, the term *β*_*θ*_ *c* ^*T*^ *β*_*θ*_ *c* is visualized as a positive constant. Although the limits of joint velocity and joint angle are not emerged into the scheme index, the range of rotated joints is still reflected in the program. Due to the deviations among initial positions of joint angles, a feedback control *r* − *f*(*θ*) is added into the motion equation of redundant manipulators to guarantee that the end-effector will back to the desired initial trajectory at last.

By analyzing and verifying the above deductions, the repeatable motion scheme (3) can be simplified as the following index:

(14) $$\begin{array}{c} \begin{array}{cc} \text{minimize} & \frac{1}{2}\;{\dot{\theta }}^{T}\dot{\theta }+\beta_{\theta }c^{T}\dot{\theta }\\ \text{subject to} & J\left(\theta \right)\dot{\theta }={\dot{r}}^{*}+k_{p}\left(r^{*}-f\left(\theta \right)\right), \end{array} \end{array}$$

where *k*_*p*_ > 0 ∈ *R* represents a feedback gain parameter. *c*=|*θ*(*t*) − *θ* ^*∗*^(0)|^*α*^sgn(*θ*(*t*) − *θ* ^*∗*^(0)). Besides, 0 < *α* < 1, *θ* ^*∗*^(0) is the initial position of joint angle vectors. The motion scheme (12) visualizes the basic kinematics of the redundant manipulators. From the optimization equation (12), joint velocity level limitations for joint angle and joint velocity are difficult to be combined with the motion scheme. The situation of exceeding the joint limits has been considered in the simulation programs.

## 4. Neural Network Solving

Considering the scheme of repeatable motion planning, we change equation (12) by using the method of Lagrange multipliers.

(15) $$\begin{array}{c} L\left(\dot{\theta }\left(t\right),\lambda \left(t\right),t\right)=\frac{{\dot{\theta }}^{T}\left(t\right)\dot{\theta }\left(t\right)}{2}+\beta_{\theta }c^{T}\dot{\theta }\left(t\right)+\lambda^{T}\left(t\right)\left(J\left(\theta \right)\dot{\theta }\left(t\right)-{\dot{r}}^{*}-k_{p}\left(r^{*}-f\left(\theta \right)\right)\right), \end{array}$$

where *λ* ^*T*^(*t*) ∈ *R* ^1×*m*^ denotes the Lagrangian-multiplier vector.

(16) $$\begin{array}{c} \frac{\partial L\left(\dot{\theta }\left(t\right),\lambda \left(t\right),t\right)}{\partial \dot{\theta }\left(t\right)}=\dot{\theta }\left(t\right)+\beta_{\theta }c+J^{T}\left(\theta \right)\lambda \left(t\right)=0,\\ \frac{\partial L\left(\dot{\theta }\left(t\right),\lambda \left(t\right),t\right)}{\partial \lambda \left(t\right)}=J\left(\theta \right)\dot{\theta }\left(t\right)-{\dot{r}}^{*}-k_{p}\left(r^{*}-f\left(\theta \right)\right)=0. \end{array}$$

The following time-varying equation (14) can be obtained:

(17) $$\begin{array}{c} W\left(t\right)y\left(t\right)=\upsilon \left(t\right). \end{array}$$

With

(18) $$\begin{array}{c} W\left(t\right)=\left[\begin{array}{cc} I & J^{T}\left(t\right)\\ J\left(t\right) & 0 \end{array}\right]\in R^{\left(n+m\right)\times \left(n+m\right)},\\ y\left(t\right)=\left[\begin{array}{c} \dot{\theta }\left(t\right)\\ \lambda \left(t\right) \end{array}\right]\in R^{n+m},\\ v\left(t\right)={\left[-\beta_{\theta }c{\dot{r}}^{*}+k_{p}\left(r^{*}-f\left(\theta \right)\right)\right]}^{T}\in R^{n+m}, \end{array}$$

where vector *I* represents the identity matrix. We may calculate the convergent error by setting

(19) $$\begin{array}{c} E\left(t\right)=W\left(t\right)y\left(t\right)-\upsilon \left(t\right). \end{array}$$

According to the neural solver (10), we get the following dynamic system equation form for trajectory planning:

(20) $$\begin{array}{c} \dot{y}=\dot{W}y+\left(W+I\right)\dot{y}-\dot{v}+\beta_{\theta }{\left|Wy-v\right|}^{\alpha }\mathrm{sgn}\left(Wy-v\right). \end{array}$$

## 5. Applications to Redundant Manipulators

In this section, two experimental examples are displayed and analyzed to illustrate the repetitiveness and finite-time convergence of the proposed motion scheme (12) for neural model solving. Comparisons on different repetitive motion schemes based on Katana6M180 manipulator are introduced to substantiate the superiority and timeliness of the proposed index for task controlling.

### 5.1. Triangle-Path Tracking Task

For this experimental simulation, the end-effector of the Katana6M180 manipulator is required to track a triangle path. During the simulation, *t* ∈ [0, *T*], the desired trajectory of the manipulator is defined as follows:

(21) $$\begin{array}{c} r_{x}^{*}=\left\{\begin{array}{cc} 0.05\;\sin^{2}\left(\frac{0.5\pi t}{3}\right), & 0\leq t\leq 3,\\ 0.05\;\sin^{2}\left(\frac{0.5\pi \left(t-3\right)}{3}\right), & 3<t\leq 6,\\ -0.1\;\sin^{2}\left(\frac{0.5\pi \left(t-6\right)}{3}\right), & 6<t\leq 9, \end{array}\right.\\ r_{y}^{*}=\left\{\begin{array}{cc} 0.05\sqrt{3}\sin^{2}\left(\frac{0.5\pi t}{3}\right), & 0\leq t\leq 3,\\ -0.05\sqrt{3}\sin^{2}\left(\frac{0.5\pi \left(t-3\right)}{3}\right), & 3<t\leq 6,\\ 0, & 6<t\leq 9, \end{array}\right.\\ r_{z}^{*}=0. \end{array}$$

**Figure 3:**
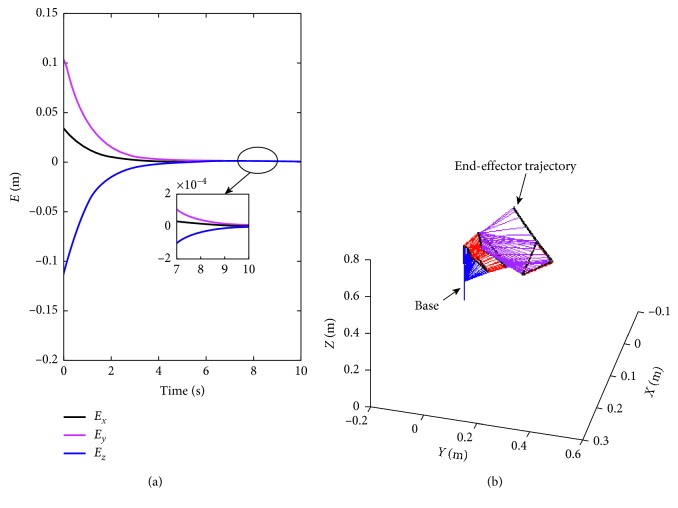
Simulation profiles for Katana6M180 manipulator synthesized with motion scheme (12).

**Figure 4 fig4:**
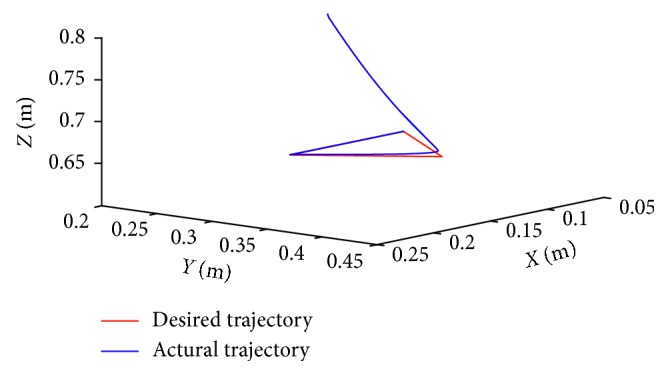
Trajectory profiles with desired path and actual path of end-effector.

**Figure 5:**
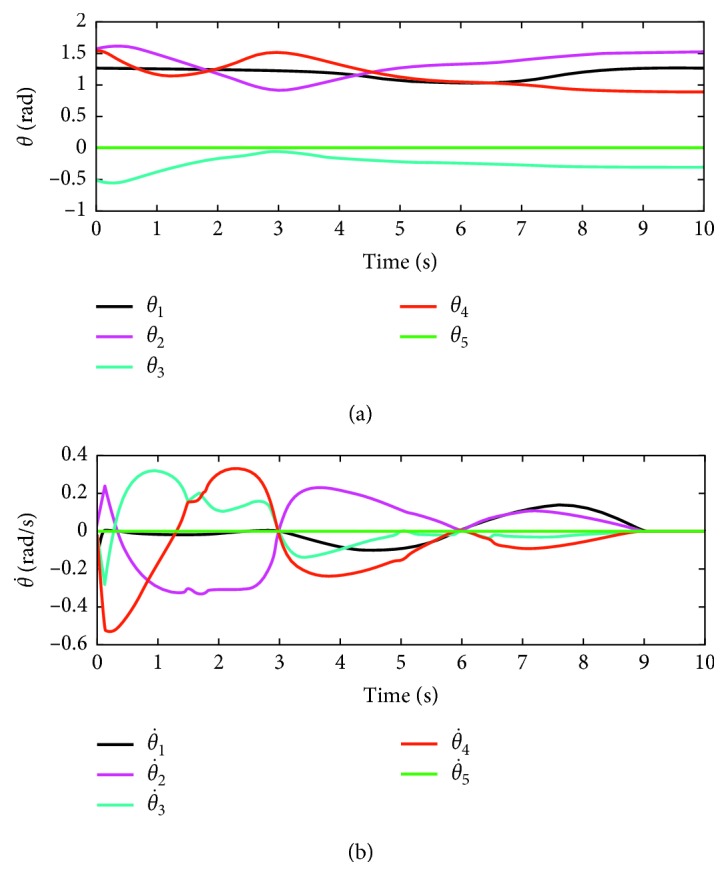
Trajectory profiles with joint angles and joint velocities of the manipulator.

The side length is assumed to be 0.18 m. The task completing time is set *T*=9 s. The desired initial position of each joint angle is set to *θ* ^*∗*^(0)=[2*π*/5, *π*/2, 0, −*π*/6, *π*/3, 0]^*T*^ rad. Considering the deviation of joint angles before tracking, the initial position of each joint angle is set to be *θ*(0)=[2*π*/5, *π*/2, 0, −*π*/6, *π*/3+0.5, 0]^*T*^ rad. Furthermore, design parameter *β*_*θ*_=1, *k*_*p*_=1, *α*=0.1. The simulation profiles are shown in Figures 3 [Fig fig4]–5. In Figure 3(b), the end-effector of K6M180 is tracking a triangle path. The end-effector comes back to its desired initial position though the initial positions are not correctly settled. In Figure 3(a), the error position of *XYZ* has converged to zero around 6 s and the convergent precision is less than 2.4 × 10^−4^ at the end of the tasks. From Figure 4 the end-effector rapidly moves to the desired motion trajectory under the repeatable motion scheme (12). That is the joint-drifting phenomenon can be remedied under the neural network solving control. With the Figure 5, the corresponding trajectory profiles of joint angles and joint velocities demonstrated the final statement of the manipulator. It is shown that all the joint angles have been returned to the desired position, and the motion velocity of each joint angle has been reduced to zero in the end. For obvious illustration, Table 3 shows the convergent errors of six joints. From Table 3, the maximum error in joint position is 3 × 10^−4^.

### 5.2. Circular-Path Tracking Task

**Figure 6:**
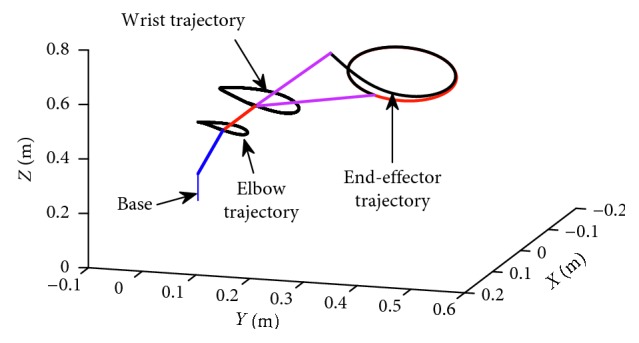
Trajectories of each joint angle to follow the circular-path tracking.

**Figure 7 fig7:**
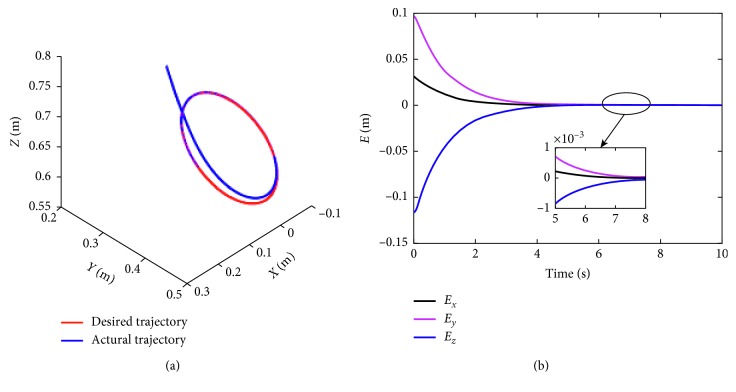
Path profiles of the end-effector when it is tracking the given path under the scheme index (12).

**Figure 8:**
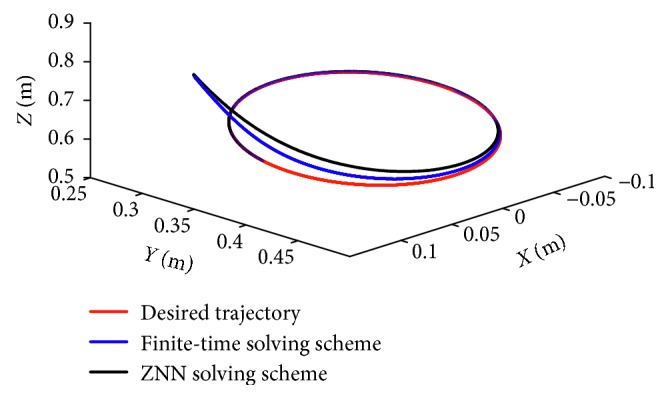
Comparison results of the end-effector when using different motion scheme index to follow a circular path.

In this part, the end-effector of the manipulator what we provide has been planned to rack a circular path. The radius of the tracking circle is set to be 0.05 m. The corresponding profiles synthesized with the motion scheme (12) under the neural solver are shown in Figures 6 [Fig fig7]–8.  Figure 6 shows the trajectory of each joint angle along the motion procedure. From Figure 6, we can find each joint has performed a closed path at last. The end-effector of the manipulators has been returned to the desired initial position although the deviations existed at first. End-effector path and position error *XYZ* are shown in Figure 7. From Figure 7(a), we can obviously discover the end-effector has chased the desired task in several time and has performed a precise circular path with time. In Figure 7(b) the convergent precision in three direction *XYZ* is about 2 × 10^−3^, which demonstrates that the task is completed well.

### 5.3. Comparisons

**Figure 9:**
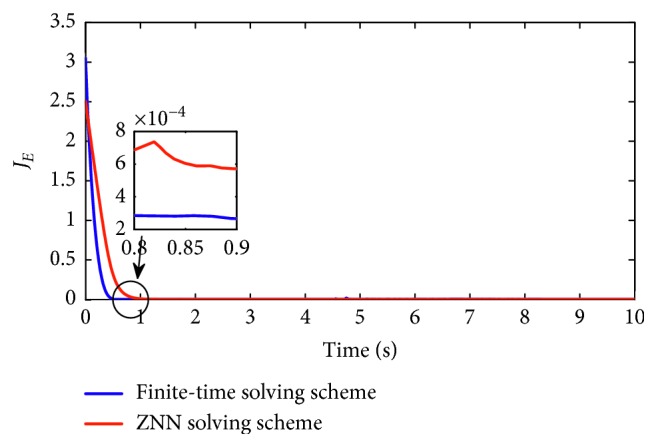
Convergent error produced by ZNN motion scheme (17) and finite-time scheme (12) for circular path.

As we discuss the repeatable motion scheme, the ZNN method formulated with the repetitive motion plan has been provided in many literatures [24, 25]. It can be generalized in velocity level as follows:

(22) $$\begin{array}{c} \begin{array}{cc} \text{minimize} & \frac{1}{2}\;{\dot{\theta }}^{T}\dot{\theta }+\mu {\left(\theta -\theta \left(0\right)\right)}^{T}\dot{\theta }\\ \text{subject to} & J\left(\theta \right)\dot{\theta }={\dot{r}}^{*}+k_{p}\left(r^{*}-f\left(\theta \right)\right), \end{array} \end{array}$$

where *μ* > 0 is the magnifying parameter to scale the convergent speed. In the motion index (17), the end-effector of the redundant manipulator can return to the initial position after executing the triangle trajectory as long as time is infinite. That means the convergent time is supposed to be infinity, which is not applicable in real-time processing. In addition, the initial position of the joint angles is assumed to be in the desired position at first, which may lead to the inaccurate convergence by manipulators. In order to illustrate the advantages of our finite-time model in repetitive motion, the comparison experiments are simulated on the same Katana6M180 manipulators. For simplifying the simulation results, we use the error norm *J*_*E*_=||*Wy* − *v*||_*F*_ to compare the convergent speed and the improved convergent precision. The corresponding results are shown in Figures 8 and 9. As seen in Figure 8, synthesized with motion scheme based on ZNN, the convergent rate is gradually catch up the red line, which is obviously slower than the finite-time solving scheme. For clear comparisons, we use *J*_*E*_ in Figure 9 and the convergent rate of finite-time solving scheme reaches to zero in 1 s. The convergent precision is about 2 × 10^−4^. For comparisons, Table 3 lists the error of the joint angles by different repeatable motion schemes. The finite-time solving scheme remedies the initial position deviation, that is *θ*_*i*_(9) − *θ*_*i*_(0) < 2.644 × 10^−5^. The deviations of the joint angle based on ZNN is around 3.744 × 10^−4^, which shows larger convergent precision in joint-drifting phenomenon.

## 6. Conclusion

In this paper, a method of solving redundant robot repetitive motion based on neural network has been proposed. The solution for manipulator motion planning not only improves the convergent precision but also accelerates the convergent rate. The motion scheme makes the end-effector of the manipulators return to the desired initial position in finite time, which is more applicable in the practical engineering fields. In addition, theoretical analysis and simulations verify the superiority and timeliness of the proposed method in solving time-varying problems, especially in motion planning of manipulators. However, robustness for the new repeatable motion scheme is not considered in the theoretical analysis, even if instable phenomena may affect the convergent rate. The effect of interference phenomenon for repetitive planning of the redundant manipulators will be considered in the future.

## Figures and Tables

**Table 1:** Joints motion range of Katana6M180 redundant manipulator.

Joint angle	Offset angle	Motion range	Unit
*θ*_1_	0	345.5	Degree
*θ*_2_	124.25	140	Degree
*θ*_3_	52.7	241.5	Degree
*θ*_4_	63.5	232	Degree
*θ*_5_	8.5	332.2	Degree

**Table 2:** D-H parameters of Katana6M180 redundant manipulator.

Link_*i*_		*a*_*i*_	*α*_*i*_	*d*_*i*_	*θ*_*i*_
1		90°	0	*l*_1_	*θ*_1_
2		0	*l*_2_	0	*θ*_2_
3		0	*l*_3_	0	*θ*_3_
4		90°	0	0	*θ*_4_
5		0	0	*l*_4_+*l*_5_	*θ*_5_

**Table 3:** Error deviations of the Katana6M180 manipulator under the different scheme (12) and scheme (17), which are solved by neural networks tracking a circular path.

Joint	Finite-time solving scheme	ZNN solving scheme
*θ*_1_(9) − *θ*_1_(0)	−0.00001982	−0.0001762
*θ*_2_(9) − *θ*_2_(0)	−0.00002644	−0.0002344
*θ*_3_(9) − *θ*_3_(0)	−0.000009387	−0.00006367
*θ*_4_(9) − *θ*_4_(0)	−0.000009656	−0.00007636
*θ*_5_(9) − *θ*_5_(0)	−8.022 × 10^−9^	−7.012 × 10^−8^

## Data Availability

The source code and source data can be provided by contacting with the corresponding author.
